# Boosting Physical Activity Among Individuals With Low Engagement Through Double-Point Incentives in a Community-Based mHealth Intervention: Retrospective Observational Study

**DOI:** 10.2196/66227

**Published:** 2025-08-21

**Authors:** Jungin Joo, Mangyeong Lee, Junghee Yoon, Hyeonjin Cho, Govind Warrier, Johannes Thrul, Juhee Cho

**Affiliations:** 1 Department of Digital Health, Samsung Advanced Institute for Health Sciences & Technology, Sungkyunkwan University, Seoul, Republic of Korea; 2 Center for Clinical Epidemiology, Samsung Medical Center, Sungkyunkwan University School of Medicine, Seoul, Republic of Korea; 3 Department of Mental Health, Johns Hopkins Bloomberg School of Public Health, Baltimore, MD, United States; 4 Department of Clinical Research Design and Evaluation, Samsung Advanced Institute for Health Sciences & Technology, Sungkyunkwan University, 717, Irwon-dong, Gangnam-gu, Seoul, 06355, Republic of Korea, 82 2-3410-1448; 5 Samsung Medical Center, Patient-Centered Outcomes Research Institute, Seoul, Republic of Korea; 6 Department of Oncology, Johns Hopkins University School of Medicine, Baltimore, MD, United States; 7 Bloomberg-Kimmel Institute for Cancer Immunotherapy and Sidney Kimmel Comprehensive Cancer Center, Johns Hopkins University School of Medicine, Baltimore, MD, United States; 8 Sidney Kimmel Comprehensive Cancer Center at Johns Hopkins, Baltimore, MD, United States; 9 Centre for Alcohol Policy Research, La Trobe University, Melbourne, Australia; 10 Department of Health, Behavior and Society, Johns Hopkins Bloomberg School of Public Health, Baltimore, MD, United States

**Keywords:** community-based interventions, physical activity, engagement, financial incentive, relapsed period, mobile phone

## Abstract

**Background:**

The administration of incentives to promote physical activity, such as the amount or timing, can vary depending on target health behaviors, research settings, intervention delivery channels, and participants’ preferences. Interventions implemented at scale necessitate the consideration of potential fiscal constraints for public health promotion. Since limited funding is a barrier to implementing community-based interventions, examining both immediate and sustained effects of temporary incentive increases on physical activity is important.

**Objective:**

This study aimed to evaluate the effect of a 1-week double-point event on increasing physical activity among low-engaged individuals in the context of a community-based mobile intervention.

**Methods:**

Using retrospective data from a Seoul Metropolitan Government mobile health (mHealth) intervention, we evaluated the effects of a 1-week double-point incentive on participants’ physical activity. During 3 registration phases from November to December 2021, a total of 50,145 individuals enrolled. Our analysis focused on the low-engaged group (n=27,833, 55.5%), who averaged fewer than 3 days per week of meeting the daily step challenge (at least 7000 steps) before the intervention. We performed a segmented regression analysis to assess changes in physical activity before and after the event. Multivariable logistic regression and Cox proportional hazards models were used to identify factors associated with improving and maintaining physical activity after starting the intervention.

**Results:**

Of 27,833 low-engaged participants, only 13.7% (n=3835) improved their physical activity. Daily challenge engagements per week increased by 2.53 times, and average daily steps increased by 1924.97 (standardized mean difference 0.55, 95% CI 0.51‐0.58). In multivariable logistic regression, older age was significantly associated with improved physical activity immediately after starting the intervention. However, 50% (1918/3835) of the improved group was likely to return to low engagement 3 weeks after the intervention ended. Older age and use of certain wearable devices were associated with maintaining physical activity after the intervention.

**Conclusions:**

Double-point incentives in the short term may serve as a cue-to-action to motivate low-engagement targets; however, they do not seem to guarantee long-term maintenance in the context of community-based mHealth interventions. Further research is needed to identify additional strategies beyond monetary incentives to sustain long-term healthy behavior.

## Introduction

Physical inactivity is the fourth leading cause of global mortality, contributing significantly to the global health burden [1]. As of 2016, a total of 27.5% of the global population was physically inactive, a prevalence that has persisted since 2001 [2]. This widespread inactivity poses health risks and results in substantial health care costs for governments and other stakeholders [3]. Achieving the recommended levels of physical activity remains challenging; for example, in 2018, a total of 39.5% of Americans aged 18 years and older did not meet the aerobic activity guidelines [4]. To combat this, many countries have implemented community-based mobile interventions, such as the Carrot Rewards program in Canada and the National Steps Challenge Season 3 in Singapore, which have successfully increased physical activity [5-7].

Despite the promising effect of community-based mobile interventions, encouraging properly those whose engagement waned during the intervention remains challenging. To address this challenge, providing financial rewards or incentives is considered a good way, demonstrating up to 2.5-fold greater effectiveness in promoting physical activity engagement compared to nonincentivized conditions [8-10]. A meta-analysis revealed that among studies only targeting physically inactive populations, financial incentives improved a mean increase of 1150 steps per day [6].

The administration of incentives, such as the amount or timing, can vary depending on target health behaviors, research settings, intervention delivery channels, and participants’ preferences [5,11-13]. Interventions implemented at scale necessitate the consideration of potential fiscal constraints for public health promotion. Thus, the incentive operation may change during the intervention administration due to business or financial decisions, and these changed rewards may impact user engagement [14]. In a longitudinal study with 55,000 users of a commercial physical activity application, reducing incentive size over time was associated with lowering user engagement [15]. Another longitudinal study revealed that adding a team-based incentive policy to the conventional individual-oriented policy contributed to improving daily step counts [16]. Additionally, a study with 1600 users reported that their physical activity decreased after reducing incentives [14].

The previous studies allowed us to examine the relationship between incentive changes and user behaviors in a real-world context [17]. However, further elucidation is needed regarding the effect of intentional, theory-based changes to incentive policies on user engagement within community-based mobile physical activity interventions. Specifically, as limited funding is a barrier to implementing community-based interventions, examining both immediate and sustained effects of temporary incentive increases on physical activity is important [18]. Therefore, our study aimed to evaluate the effect of a 1-week double-point event on increasing physical activity among low-engaged individuals in the context of a community-based mobile intervention. We also identified which participants increased their activity due to the event and assessed how long these improvements lasted.

## Methods

### Study Population

This retrospective cohort study used data from “On Seoul, Health On (OSHO),” a 9-month community-based mobile health (mHealth) intervention program organized by the Seoul Metropolitan Government from November 2021 to July 2022. The analysis focused on a subset of participants who were categorized into the low-engaged group in terms of physical activity during the program’s initial phase (1 month). The low-engaged group was defined as individuals who engaged in the daily step count challenge (completing at least 7000 steps) for an average of less than 3 days per week. Several studies suggest that taking 7000 steps per day is a minimum recommended level of physical activity to reduce the risk of morbidity or mortality in adults [19-21]. Moreover, the government aimed to facilitate the participants’ self-efficacy by setting achievable goals for a wide range of age groups [22,23]. Additionally, setting it to 7000 steps per day can be reasonable considering that the average number of steps for Koreans is 5755 [24], and it is usually recommended to increase the target setting by about 10%‐15% [6]. A total of 50,145 participants registered for the program, and 27,833 (55.5%) individuals in the low-engaged group during the initial phase of the program were included in this study (Figure S1 in Multimedia Appendix 1).

### Description of the OSHO Program

The OSHO program aimed to enhance physical activity levels of individuals in Seoul, South Korea. Eligibility for the program required participants to be adults aged 19‐64 years, residing or working in Seoul, and owning a mobile device compatible with at least Android (version 5.0; Google LLC) or iOS (version 10.0; Apple). Recruitment was conducted through the program’s official homepage, media coverage, and advertisements in subway stations. During 3 registration phases spanning November to December 2021, a total of 50,145 individuals registered to participate. Upon registration, participants were provided with 1 of 4 different wearable devices free and asked to install a mobile app for the program. All wearable devices measured steps in real time, but the shape (band-like or watch-like) and the availability of additional functions (body composition, heart rate, etc) varied depending on the brand. Detailed specifications of each wearable brand are presented in the supplementary materials (Table S1 and Figure S2 in Multimedia Appendix 1). The mobile app enabled participants to monitor their step counts as recorded by the wearable device. To encourage participation, individuals received 200 points, convertible to an equivalent amount of cash (KR ₩200, approximately US $0.20), for every 7000 steps or more per day. The points could be redeemed to promote their health at pharmacies, sports facilities, and other designated health care businesses.

### “Spring Walk Campaign”: Additional Financial Incentive Event

Approximately 4 months after the start of the program, the “Spring Walk Campaign” (SWC) was introduced as a special 1-week event, starting on March 21, 2022. The SWC was intended to encourage physical activity among participants who remained inactive during the initial intervention by providing additional financial incentives. The SWC was advertised to OSHO participants through the mobile app and the official website. During the event, those who engaged in the daily step challenge were awarded double points (400 points) to encourage physical activity. We anticipated that the SWC, a temporary incentive increase, could motivate the low-engaged participants based on a behavioral mechanism that combines “loss aversion” and “scarcity effects” [25]. Although the intervention offered additional rewards rather than removing existing ones, which is not a classical use of loss aversion, the limited-time nature may have triggered similar urgency effects associated with perceived opportunity costs.

### Outcomes

The primary outcome was the immediate change in physical activity during the SWC event. Physical activity was evaluated using steps counted by wearable devices. Individuals who succeeded in the daily step challenge (>7000 steps) for at least 3 days a week were considered the improved group. The secondary outcome was the maintenance of improved physical activity among participants who transitioned to the improved group during the SWC after the removal of additional incentives.

At the time of registration for this program, sociodemographic information, including sex, age, and occupation, was assessed. The BMI and comorbidities were also assessed. Physical activity levels at enrollment were assessed using the International Physical Activity Questionnaire-7 Short Form [26]. This measure is a self-administered tool that focuses on the frequency and duration of walking, moderate to vigorous intensity activities, and sedentary time over the past 7 days. In this study, we adapted the instrument by using 6 items while excluding 1 item that assessed sedentary behavior. Responses were converted to metabolic equivalent of task (MET)–minutes per week, an objective metric that quantifies the energy cost of physical activities [27]. One MET is defined as the amount of oxygen consumed while sitting at rest and equals 3.5 mL O₂ per kg body weight×min [28]. Based on MET-min/wk values, participants’ physical activities were categorized into 3 levels—low, moderate, and high. The criteria for these categorizations are detailed in Table S2 in Multimedia Appendix 1. Step count data recorded in real time through wearable devices were summarized and shared as daily cumulative figures. All distributed devices were manufactured by Korean companies and had obtained KC (Korea Certification) for product safety and qualification testing. Only authorized personnel had access to the data on the OSHO administration server, and the research team obtained deidentified data.

### Statistical Analyses

Descriptive analyses were performed to summarize participants’ baseline characteristics. Significance tests were conducted using the chi-square test (for categorical variables) or *t* test (for continuous variables), and the standardized mean difference (SMD) was estimated to present the magnitude of the difference between the 2 groups, considering this study’s population size.

We performed a segmented regression to analyze changes in daily user engagement trajectories before and after the SWC. An equation of the regression model was considered to define levels (eg, y-intercepts) and slopes for each time segment (before vs after starting the SWC). Time started from 0, and the intervention was coded as 0 (before starting the SWC) and 1 (after starting the SWC). Time since the intervention was measured in days post the SWC. The model was fitted using the restricted maximum likelihood method, including the autoregressive error term. The autocorrelation value was calculated using the Durbin-Watson test. After fitting, we presented a graphical depiction of the data points and trend lines. Multivariable logistic regression was used to identify the factors associated with improved physical activity (the improved group) after the event. This model was adjusted for potential confounders, including age, sex, BMI, physical activity level at enrollment, chronic diseases, OS type, and wearable device type. The inclusion of OS type was due to its potential to reflect individual behavior disposition [29] or varying motivations for healthy behavior change among users [30]. Additionally, we considered that heterogeneity across wearable brands may result in different user experiences and satisfaction with the OSHO program [31,32]. This was because certain brands had high dropout rates in the exploratory analysis, which may be a confounder or attrition bias (Table S3 in Multimedia Appendix 1). Technological features are also known to influence user engagement [33]. Kaplan-Meier survival curves were plotted to estimate the time to the first occurrence of relapse to the low engagement level in the improved group, with weekly time points. Differences between curves were assessed using the log-rank test. All statistical analyses were conducted using R (version 4.3.1; R Foundation for Statistical Computing). Statistical significance was defined as a 95% CI and a *P* value of <.05.

### Ethical Considerations

This study was approved by the institutional review board of the Samsung Medical Center (SMC 2022-07-073). All individuals who participated in the OSHO program provided informed consent for the program registration. However, our research uses retrospective data containing anonymized personal information and has received a waiver of additional informed consent for the analyses from the participants. Participants received 200 points (equivalent to 200 Korean Won or approximately USD 0.20) for each day they achieved 7,000 steps or more. These points could be redeemed at pharmacies, sports facilities, and other designated health-related businesses.

## Results

### Comparison of Demographic Characteristics Between Those With Improved Physical Activity During the SWC and Those With No Improvement

Of 27,833 participants included in the analysis, 3835 (13.7%) showed improved physical activity during the SWC (Figure S1 in Multimedia Appendix 1). The improved group members were older (39.4 vs 37.4 years, SMD=0.194) and especially included more adults aged 40 years and over, compared to the nonimproved group (SMD=0.215, Table 1). The BMI (SMD=0.031) and physical activity level at baseline (SMD=0.054) were similar between the 2 groups.

**Table 1.** Difference in demographic characteristics by improved physical activity during the Spring Walk Campaign.

	Overall (N=27,833)	Improved physical activity during the Spring Walk Campaign		Standardized mean difference
		Yes (n=3835)	No (n=23,998)	
Age (years), mean (SD)	37.7 (10.3)	39.4 (10.2)	37.4 (10.3)	0.194
Age groups (years), n (%)				
20s	7730 (27.8)	797 (20.8)	6933 (28.9)	0.215
30s	9214 (33.1)	1241 (32.4)	7973 (33.2)	
40s	7034 (25.3)	1168 (30.5)	5866 (24.4)	
50s	3073 (11)	498 (13)	2575 (10.7)	
60s	782 (2.8)	131 (3.4)	651 (2.7)	
Sex (female)	18,470 (66.4)	2644 (68.9)	15,826 (65.9)	0.064
BMI (kg/m^2^), mean (SD)	23.9 (18.4)	23.8 (3.7)	23.9 (19.6)	0.013
BMI categories (kg/m^2^), n (%)				
Underweight	1433 (5.1)	181 (4.7)	1252 (5.2)	0.031
Normal	12,272 (44.1)	1671 (43.6)	10,601 (44.2)	
Overweight	5525 (19.9)	790 (20.6)	4735 (19.7)	
Obese	8603 (30.9)	1193 (31.1)	7410 (30.9)	
Physical activity level, n (%)				0.054
Inactive	16,028 (57.6)	2132 (55.6)	13,896 (57.9)	
Minimally active	6586 (23.7)	981 (25.6)	5605 (23.4)	
High active	5219 (18.8)	722 (18.8)	4497 (18.7)	
Chronic diseases, yes, n (%)	5016 (18)	719 (18.7)	4297 (17.9)	0.022
OS type (%)^a^, n (%)				0.137
iOS	9393 (33.9)	1085 (28.4)	8308 (34.8)	
Android	18,322 (66.1)	2735 (71.6)	15,587 (65.2)	
Wearable device type (%), n (%)				0.154
A	16,297 (58.6)	2424 (63.2)	13,873 (57.8)	
B	5621 (20.2)	751 (19.6)	4870 (20.3)	
C	1156 (4.2)	177 (4.6)	979 (4.1)	
D	4759 (17.1)	483 (12.6)	4276 (17.8)	

a N= 27,715, eliminated 118 missing values.

### Factors Associated With Moving to the Improved Group During the SWC

In multivariable logistic regression, participants in their aged (years) 30s (adjusted odds ratio [aOR] 1.40, 95% CI 1.27‐1.54), 40s (aOR 1.79, 95% CI 1.62‐1.97), 50s (aOR 1.73, 95% CI 1.53‐1.95), and 60s (aOR 1.82, 95% CI 1.48‐2.23) were more likely to show improved physical activity during the SWC (Table 2). Additionally, those who had minimal physical activity levels assessed using the International Physical Activity Questionnaire-7 Short Form at baseline (aOR 1.16, 95% CI 1.07‐1.26) or used android-powered smartphones compared to iOS (aOR 1.20, 95% CI 1.11‐1.30) were more likely to show improved physical activity. However, those who received wearable B (aOR 0.89, 95% CI 0.81‐0.97) or wearable D (aOR 0.63, 95% CI 0.57‐0.70) were less likely to show improved physical activity.

**Table 2.** Factors associated with improved physical activity during the Spring Walk Campaign (N=27,833).

	Adjusted OR (95% CI)^a^
Age (years)	
20s	*Reference*
30s	1.40 (1.27-1.54)
40s	1.79 (1.62-1.97)
50s	1.73 (1.53-1.95)
60s	1.82 (1.48-2.23)
Sex (female)	1.23 (1.14-1.33)
BMI (kg/m^2^)	
Underweight	*Reference*
Normal	1.05 (0.89-1.24)
Overweight	1.10 (0.92-1.32)
Obese	1.09 (0.92-1.30)
Physical activity level	
Inactive	*Reference*
Minimally active	1.16 (1.07-1.26)
High active	1.08 (0.99-1.19)
Chronic diseases (yes)	0.93 (0.84-1.02)
OS type (%)^b^	
iOS	*Reference*
Android	1.20 (1.11-1.30)
Wearable device type (%)	
A	*Reference*
B	0.89 (0.81-0.97)
C	1.00 (0.84-1.18)
D	0.63 (0.57-0.70)

a Adjusted Model included age group, sex, BMI, physical activity level, chronic diseases, type of operating system, and wearable device type.

b N=27,715, eliminated 118 missing values.

### Immediate Changes in Physical Activity After Starting the SWC

**Figure 1.**
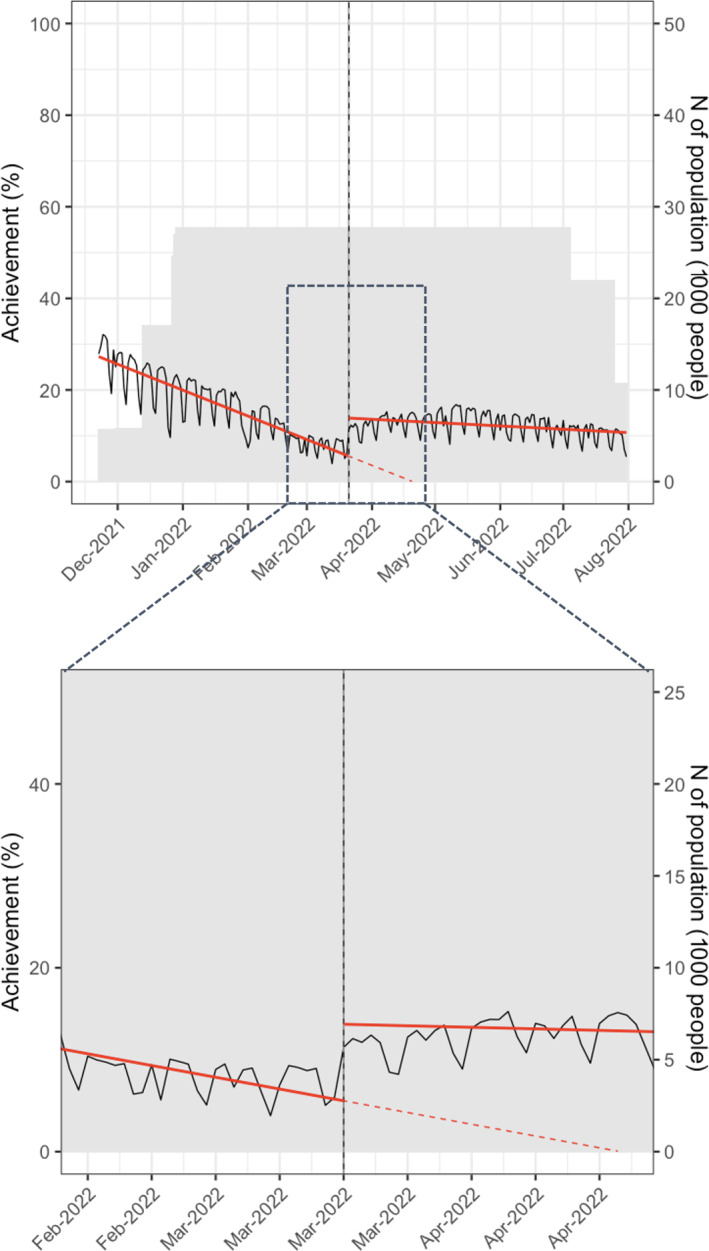
Graphical depiction of interrupted time series analysis of physical activity engagement before and after the Spring Walk Campaign.

Daily activity challenge achievement rate (%) tended to decrease by 0.18 units per day at 27.45 (Figure 1). After starting the SWC, the achievement rate immediately increased by 8.16 (*P*<.001). Especially, those with improved physical activity during the SWC increased their weekly engagement from an average of 1.76 to 4.29 times (SMD=1.81, 95% CI 1.76‐1.86, Table 3). Concerning average step count, there was an increase of 1924.97 steps in those with improved physical activity during the SWC (SMD=0.55, 95% CI 0.51‐0.58).

**Table 3.** Changes in the number of daily challenge engagement per week and average daily step counts before and during the Spring Walk Campaign.

		Before the Spring Walk Campaign, mean (SD)	During the Spring Walk Campaign, mean (SD)	Mean difference	Standardized mean difference (95% CI)
The number of daily challenge engagements per week					
Overall		0.57 (0.87)	0.77 (1.56)	0.20	0.17 (0.16 to 0.18)
Improved group (N=3835)		1.76 (0.83)	4.29 (1.22)	2.53	1.81 (1.76 to 1.86)
Nonimproved group (N=23,998)		0.38 (0.71)	0.21 (0.54)	−0.17	−0.26 (−0.27 to –0.25)
Average step counts per day					
Overall		2857.22 (3724.84)	2243.80 (3714.70)	−613.42	−0.18 (−0.19 to –0.17)
Improved group (N=3835)		6769.13 (3640.74)	8694.10 (2857.22)	1924.97	0.55 (0.51 to 0.58)
Nonimproved group (N=23,998)		2232.08 (3337.30)	1213.01 (2643.52)	−1019.07	−0.32 (−0.34 to –0.31)

### Sustaining Improved Physical Activity After the SWC Ended

**Figure 2.**
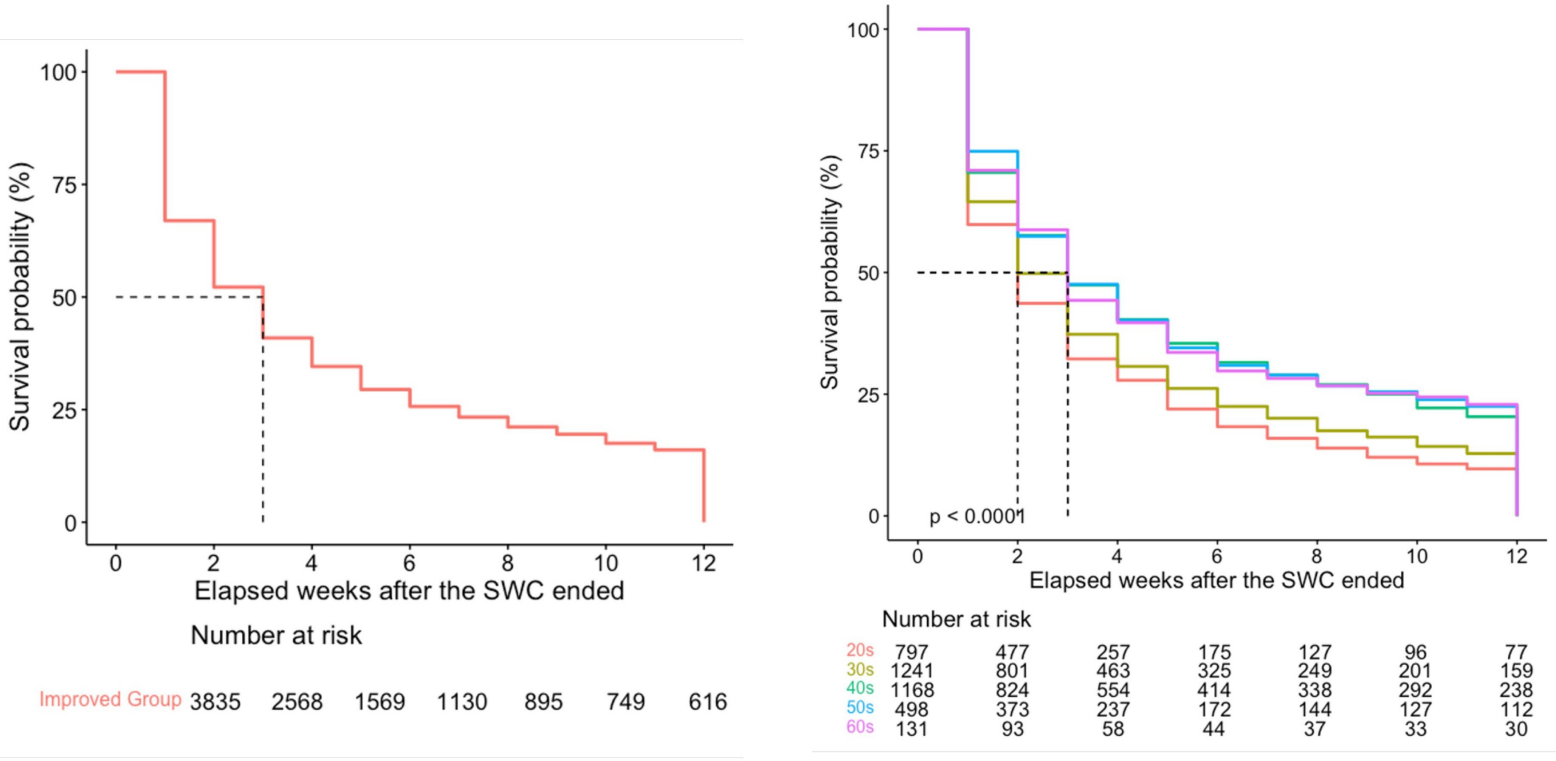
Kaplan-Meier curves for time to return to low engagement in physical activity after the Spring Walk Campaign ended among the improved group. SWC: Spring Walk Campaign.

After starting the SWC, the decreasing slope of the completion rates improved from −0.18 to −0.02 (*P*<.001, Figure 1). The Kaplan-Meier curve showed that half of the improved group were likely to return to low engagement in physical activity 3 weeks after the SWC ended (Figure 2). In the multivariable Cox proportional hazards model, participants in their aged (years) 20s (adjusted hazard ratio [aHR] 1.44, 95% CI 1.19‐1.75) or 30s (aHR 1.27, 95% CI 1.05‐1.53) had a higher risk of returning to low engagement in physical activity after the SWC ended (Table 4). Moreover, users of wearable B (aHR 1.12, 95% CI 1.03‐1.21) and wearable D (aHR 1.15, 95% CI 1.04‐1.27) had a greater risk of returning to low engagement compared to users of wearable A.

**Table 4.** Factors associated with relapse to low physical activity engagement after the Spring Walk Campaign among participants with improved physical activity during the Spring Walk Campaign^a^.

	Crude hazard ratio (95% CI)	Adjusted^b^ hazard ratio (95% CI)^c^^,d^
Age group (years)		
20s	1.43 (1.19-1.72)	1.44 (1.19-1.75)
30s	1.26 (1.05-1.51)	1.27 (1.05-1.53)
40s	1.02 (0.85-1.23)	1.03 (0.86-1.24)
50s	0.99 (0.82-1.20)	1.00 (0.82-1.21)
60s	*Reference*	*Reference*
Sex (female)	0.98 (0.91-1.07)	0.99 (0.91-1.08)
BMI (kg/m^2^), mean (SD)	1.04 (0.97-1.12)	1.04 (0.96-1.11)
Underweight	*Reference*	*Reference*
Normal	0.92 (0.79-1.07)	0.98 (0.84-1.14)
Overweight	0.96 (0.81-1.12)	1.05 (0.89-1.24)
Obese	0.96 (0.82-1.12)	1.05 (0.89-1.24)
Physical activity level		
Inactive	*Reference*	*Reference*
Minimally active	0.97 (0.90-1.04)	0.98 (0.91-1.06)
High active	0.99 (0.91-1.07)	1.01 (0.92-1.10)
Chronic diseases (yes)	0.93 (0.85-1.00)	1.02 (0.93-1.12)
OS type (%)^d^		
iOS	*Reference*	*Reference*
Android	0.89 (0.83-0.95)	0.96 (0.89-1.03)
Wearable device type (%)		
A	*Reference*	*Reference*
B	1.12 (1.03-1.21)	1.12 (1.03-1.21)
C	0.89 (0.76-1.04)	0.93 (0.80-1.08)
D	1.11 (1.01-1.23)	1.15 (1.04-1.27)

a The improved group (N1=3820).

b Eliminated 15 missing values.

c Crude model represents the crude hazard ratio (95% CI).

d Adjusted Model included age group, sex, BMI, physical activity level, chronic diseases, OS type, and wearable device type.

## Discussion

### Principal Findings

In this study, we evaluated the effect of a 1-week double-point event on increasing physical activity among low-engaged individuals in the context of a community-based mobile intervention. We also identified which participants increased their activity due to the event and assessed how long these improvements lasted. We found that 14% (n*=*3835) of those who were in the low-engaged group with the intervention improved their physical activity during the SWC. However, the effect diminished in 50% (1918/3835) of the participants within 3 weeks, with those in their 20s and 30s showing a more rapid return to preintervention levels.

### Immediate Effects of a 1-Week Double-Point Incentive Event on Change of Physical Activity

In our study, the OSHO program provided a stream of small-size incentives (US $0.20 per day) for achieving the daily physical activity challenge. However, participants’ physical activity or engagement with the program declined over time. We found that temporarily increasing incentives could help improve intervention engagement, potentially leading to physical activity improvement. Immediately after starting the SWC, the achievement rate of the daily activity challenge increased by 8.16%. In the low-engaged group of 27,833 people, approximately 14% (n=3835) showed an improvement in their physical activity during the SWC. In general, larger absolute amounts of financial incentives tend to ensure greater user engagement with mobile interventions [5,8]. However, recent evidence highlights that considering how to design an incentive structure is more important than securing a larger amount of incentives. A study using real-world data from 54,817 Canadians found that decreasing incentives over time can lead to lower user engagement in mobile health programs [15]. Another real-world study found that policy change toward increasing the uncertainty of obtaining incentives led to decreased physical activity among users [14]. A randomized controlled trial in which the total amount of incentives was the same but the structure was different found that a constant incentive rate was more effective than increasing or decreasing rates over time [34]. Therefore, considering our research and previous studies, it would be necessary to develop and evaluate effective incentive policies to increase user participation. However, since the overall effect size of SWC was low (SMD=0.17) and certain participants responded to it, multiple incentive structures should be established within an intervention [35].

### Lasting Effects of a 1-Week Double-Point Incentive Event on Change of Physical Activity

Although 14% (n=3835) of the low-engaged group became more active during the SWC, 50% (1918/3835) of them relapsed into the low-engaged group within 3 weeks after the event. This suggests that the temporary incentive increase does not ensure long-term effects on physical activity. Participants may feel that the end of SWC was akin to a withdrawal of an incentive. Though attitudinal assessment of participants was not a primary focus of this investigation, study of participants’ attitudes toward the SWC will be important in future initiatives.

Eventually, this may lead to lowering their motivation. In previous studies, financial incentives rarely led to long-term behavior change; particularly, people tended to revert to their initial behaviors soon after the incentives stopped [36]. A large randomized clinical trial evaluating the effects of a fitness tracker and various incentive structures on physical activity found that those who received cash incentives were more physically active than the control group for 6 months [5]. However, the effect diminished over the 6 months after the incentives ended. A meta-analysis also indicated that only 4 of the 18 studies exhibited positive effects following the end of the interventions. These interventions include various types of incentives, such as immediate rewards, team-based incentives, loss-framed incentives, etc, based on a randomized controlled trial [6]. In general, monetary incentives could encourage extrinsic motivation while discouraging intrinsic motivation [37]. Therefore, strategies are needed that can quickly convert extrinsic rewards provided within a temporary event into intrinsic motivation [36-38]. For instance, combining wearable technology with regular counseling produced higher engagement rates, indicating that integrating financial incentives with personalized support and feedback may be a good option [39].

### Impact of Age and Technology-Related Factors on Physical Activity Changes

In our study, we found that the older age group had greater responsiveness to the SWC and were more likely to maintain their improved physical activity. These findings are supported by previous studies that have noted greater participation among older adults in physical activity interventions [8,40]. According to a study using combined data from 8 studies with 100,000 participants, older age was a significant factor associated with retention in remote digital health studies [41]. Another longitudinal study with 1000 participants found that older age was positively associated with consistent and continued use of mobile health apps after becoming a user [42]. A possible reason is that older people are relatively more responsive to health care services, so their satisfaction and perceived benefits from mHealth may have been well maintained [43]. This may reflect the older population’s greater temporal flexibility compared to younger individuals constrained by occupational commitments. Another possible explanation may be that older people placed a higher value on the use of incentives provided in our study. According to the administrative manual of the OSHO program, the places where the points could be redeemed were limited to pharmacies, sports facilities, and other designated health care businesses to improve their health. This may not have been appealing to younger participants. Indeed, an exploratory study with Israeli adults (n=379) aged 20‐89 years reveals that while participants in the young age group (ages 20‐39 years) rated 4 factors—affiliation, competition, appearance, and mastery—as more important. In contrast, the older age group (ages 61‐89 years) rated health benefits as more important than the other age groups [44]. For younger participants, strategies such as group exercises, challenges, and sharing achievements on social media could be effective to encourage intrinsic motivation. Hence, it would be necessary to consider tailored strategies to balance extrinsic and intrinsic motivation for each age group [38].

Although we did not directly evaluate the participants’ user experience or investigate technological problems, it is possible that participants had negative experiences with certain brands of wearable devices. The Seoul Metropolitan Government tried to distribute devices with similar specifications, but our exploratory analysis discovered that brands A and B had higher dropout rates. This suggests that compatibility issues with the OSHO app or other technological problems with the devices may have negatively affected the user experience. In fact, heterogeneity between devices and systems for a single service can easily create compatibility problems [45]. This may have affected engagement with the intervention and, eventually, motivation for physical activity [33]. Therefore, rather than solely considering incentives as a means of increasing physical activity, it is important to incorporate design and administrative considerations that ensure consistent performance and user experience across platforms and devices.

### Limitations

Limitations of the current study may include confounding by unmeasured variables. The Seoul Metropolitan Government did not measure baseline health conditions such as weight, BMI, or average steps before starting this promotion. We applied retrospective analyses to evaluate a specific intervention with a large population in a real-world setting where experimental manipulations are not feasible. However, this approach may also have limitations in clearly identifying the causal effects of the intervention due to unclear or selective exposure to the intervention. To better validate the efficacy of additional financial incentives, conducting a randomized controlled trial will be needed. Behavioral aspects related to physical activity, which could impact participants’ motivation in the SWC, were also not considered. The use of wearable devices or smartphone apps to track physical activity may result in technological limitations or inaccuracies in data collection. The OSHO program provided all participants with wearable devices to monitor daily activities. However, there may still be a possibility of underestimation of daily steps if participants did not consistently wear the device. Additionally, not all participants had equal access to or familiarity with the technology, potentially affecting the results. Moreover, this study may not have adequately measured or addressed variations in participant engagement and adherence to the intervention, which could have affected the outcomes. Future research should explore methods to improve and measure engagement to enhance the effectiveness of similar interventions. Finally, the sample consisted solely of Seoul citizens, where the walkability index ranks among the top 10 cities in the world [46]. The generalizability of our findings could vary depending on the infrastructure of a given location. Future studies should replicate and extend the current findings using more diverse and representative samples from different regions to enhance the generalizability of the results.

### Conclusions

In conclusion, we found that the double-point incentive motivated only a small subset of participants, leading to significant short-term increases in physical activity. However, only a small proportion of participants maintained these behavioral changes, indicating that sustaining such changes typically requires longer or repeated interventions and personalized strategies to enhance both engagement and long-term adherence. Financial incentives alone may not be sufficient to promote sustained physical activity. Other factors, such as personalized support, behavioral change techniques, and environmental modifications, should be considered to effectively enhance physical activity levels. Integrating these elements can provide a more comprehensive approach to motivate individuals and sustain healthy behaviors.

## Supplementary material

10.2196/66227 Multimedia Appendix 1 Additional figures’ and tables’ final edits: Sankey plot, wearable device description and specifications, IPAQ-7 Short Form Scoring protocol, and devices’ attrition rate. IPAQ-7: International Physical Activity Questionnaire - Short Form.

## Abbreviations

aHR: adjusted hazard ratio
aOR: adjusted odds ratio
KC: Korea Certification
MET: metabolic equivalent of task
mHealth: mobile health
OSHO: On Seoul, Health On
SMD: standardized mean difference
SWC: Spring Walk Campaign
