# Supplementary material for: Boosting Physical Activity Among Individuals With Low Engagement Through Double-Point Incentives in a Community-Based mHealth Intervention: Retrospective Observational Study
Source: JMIR Mhealth Uhealth. 2025 Aug 21;13:e66227. doi: 10.2196/66227 (PMC12370261; doi:10.2196/66227)
Supplement: Multimedia Appendix 1 — Additional figures’ and tables’ final edits: Sankey plot, wearable device description and specifications, IPAQ-7 Short Form Scoring protocol, and devices’ attrition rate. IPAQ-7: International Physical Activity Questionnaire - Short Form. [file mhealth-v13-e66227-s001.docx]

**Additional materials**

**Figure S1.** Sankey plot for LE group in pre-intervention.

**Figure S2.** Physical appearance of each wearable device.

**Table S1.** Technical specifications of each wearable device.

**Table S2.** IPAQ-7 Short Form Scoring protocol.

**Table S3.** Attrition rate by device.


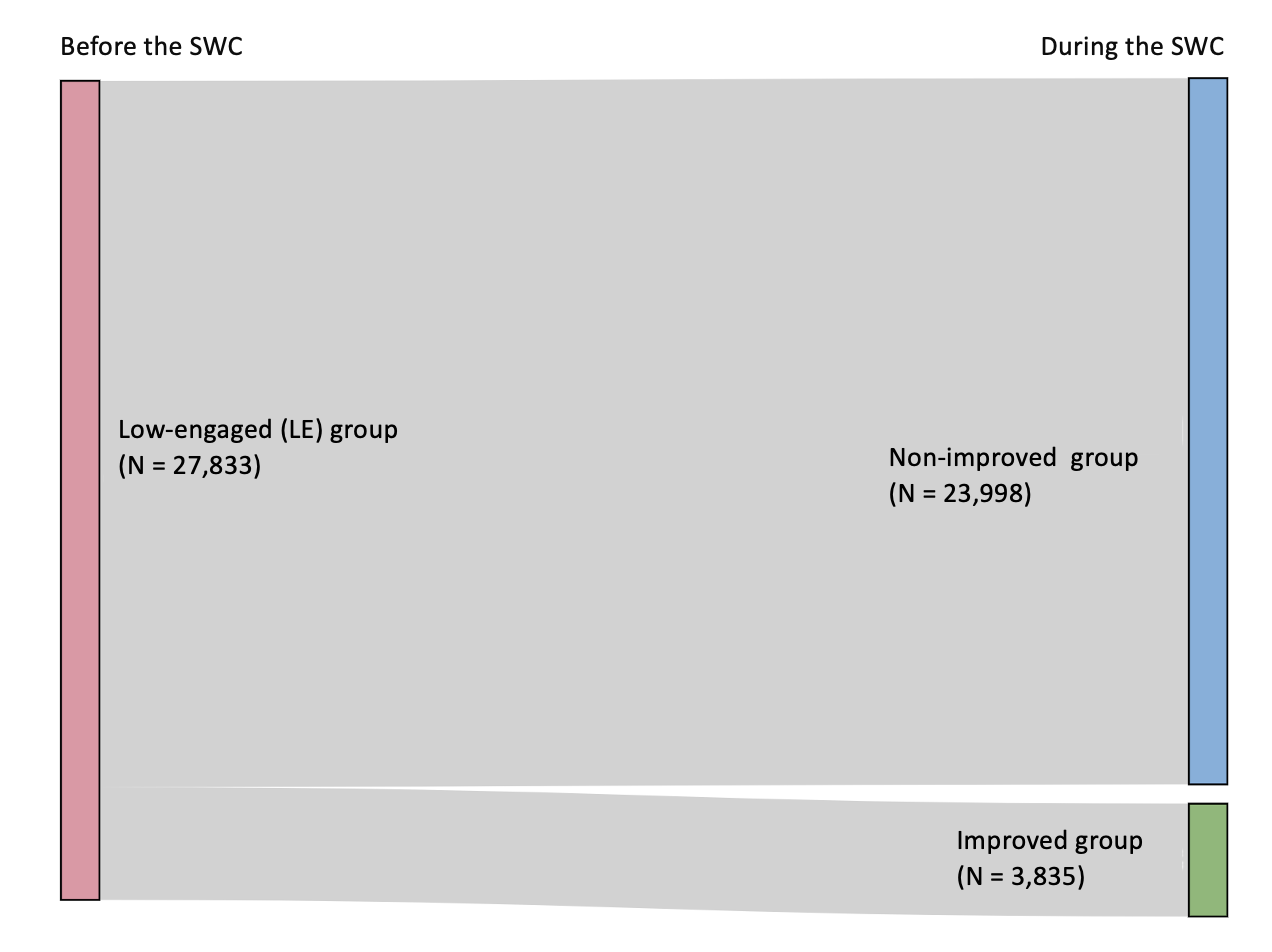


**Figure S1.** Sankey plot for LE group in pre-intervention.


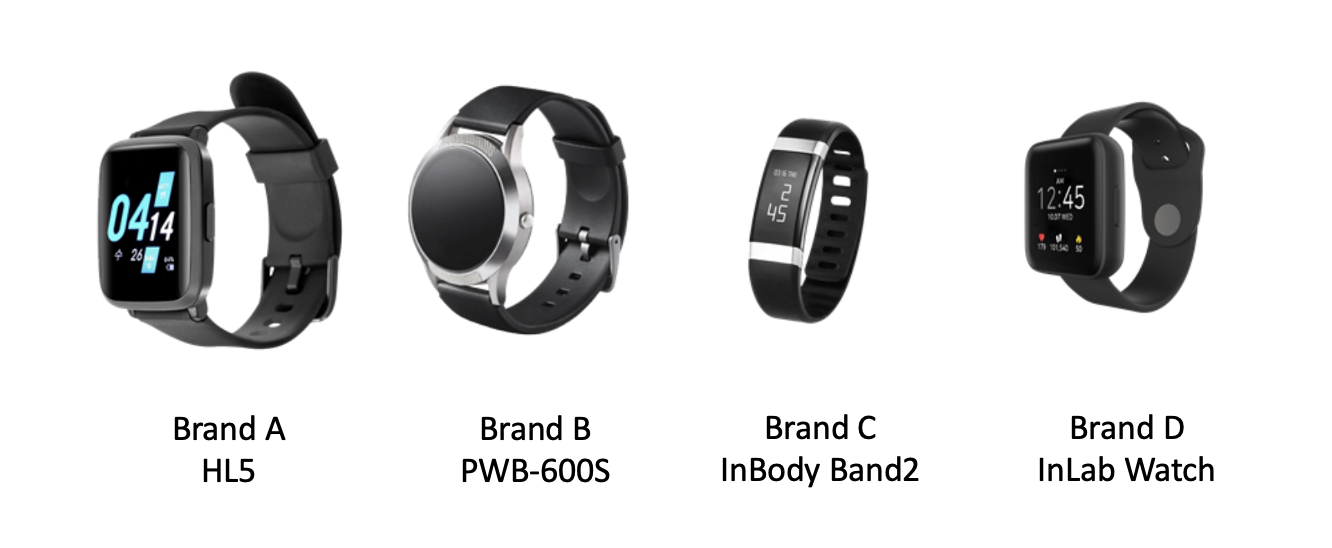


**Figure S2.** Physical appearance of each wearable device.

**Table S1.** Technical specifications of each wearable device.

| **Brands** | **Shape** | **Step count** | **Heart rate** | **Body  composition** | **Weight** | **Released year** |
| --- | --- | --- | --- | --- | --- | --- |
| Wearable A | Watch-like | X | X |  | 38g | 2021 |
| Wearable B | Watch-like | X | X |  | 38.5g | 2020 |
| Wearable C | Watch-like | X | X |  | 35g | 2019 |
| Wearable D | Band-like | X |  | X | 25g | 2017 |

**Table S2.** IPAQ-7 Short Form Scoring protocol.

| Category | Scoring protocol^a^ |
| --- | --- |
| **Category 3: High** | A separate category labelled ‘high’ can be computed to describe higher levels of participation.  The two criteria for classification as ‘high’ are:  a) vigorous-intensity activity on at least 3 days achieving a minimum Total physical activity of at least 1500 MET-minutes/week  OR  b) 7 or more days of any combination of walking, moderate-intensity or vigorous-intensity activities achieving a minimum Total physical activity^b^ of at least 3000 MET-minutes/week. |
| **Category 2: Moderate** | The pattern of activity to be classified as ‘moderate’ is either of the following criteria:  a) 3 or more days of vigorous-intensity activity of at least 20 minutes per day  OR  b) 5 or more days of moderate-intensity activity and/or walking of at least 30 minutes per day  OR  c) 5 or more days of any combination of walking, moderate-intensity or vigorous intensity activities achieving a minimum Total physical activity of at least 600 MET-minutes/week. |
| **Category 1: Low** | This is the lowest level of physical activity. Those individuals who not meet criteria for Categories 2 or 3 are considered to have a ‘low’ physical activity level. |

^a^Guidelines for data processing and analysis of the International Physical Activity Questionnaire (IPAQ) – Short and long forms [1].

^b^Total MET-min/week = (Walking min/day × Days × 3.3) + (Moderate min/day × Days × 4.0) + (Vigorous min/day × Days × 8.0).

**Table S3.** Dropout rate by device.

| **Brands^a^ (***n* **=** 50,137) **^b^** | **Dropout rate^c^ (%)** |
| --- | --- |
| Wearable A (*n* = 30,127) | 60.1% |
| Wearable B (*n* = 10,004) | 20.0% |
| Wearable C (*n* = 2,501) | 5.0% |
| Wearable D (*n* = 7,505) | 15.0% |

^a^The number of participants assigned to each device based on the total number of participants in the program.

^b^N = 50,137; 8 with missing information were eliminated.

^c^Dropout rate was defined as a participant who did not enter any data for four consecutive weeks.

**References**

1. IPAQ Research Committee. Guidelines for data processing and analysis of the International Physical Activity Questionnaire (IPAQ) – Short and long forms. 2005.Available from: <https://sites.google.com/view/ipaq/home?authuser=0>.
